# Resistance Mutations and CTL Epitopes in Archived HIV-1 DNA of Patients on Antiviral Treatment: Toward a New Concept of Vaccine

**DOI:** 10.1371/journal.pone.0069029

**Published:** 2013-07-09

**Authors:** Jennifer Papuchon, Patricia Pinson, Estibaliz Lazaro, Sandrine Reigadas, Gwendaline Guidicelli, Jean-Luc Taupin, Didier Neau, Hervé Fleury, The Provir/Latitude 45 project

**Affiliations:** 1 Laboratoire de Virologie, Hôpital Pellegrin, CHU de Bordeaux et CNRS UMR 5234 (MFP), Université de Bordeaux Segalen, Bordeaux, France; 2 Service de Médecine Interne, Hôpital du Haut Léveque, CHU de Bordeaux, Pessac, France; 3 Laboratoire d’Immunologie et Immuno-Génétique, Hôpital Pellegrin, CHU de Bordeaux et CNRS UMR 5164, Bordeaux, France; 4 Service des Maladies Infectieuses et Tropicales, Hôpital Pellegrin, CHU de Bordeaux et CNRS UMR 5234, Université de Bordeaux Segalen, Bordeaux, France; University of Missouri, United States of America

## Abstract

Eleven patients responding successfully to first-line antiretroviral therapy (ART) were investigated for proviral drug resistance mutations (DRMs) in RT by ultra-deep pyrosequencing (UDPS). After molecular typing of the class I alleles A and B, the CTL epitopes in the Gag, Nef and Pol regions of the provirus were sequenced and compared to the reference HXB2 HIV-1 epitopes. They were then matched with the HLA alleles with determination of theoretical affinity (TA). For 3 patients, the results could be compared with an RNA sample of the circulating virus at initiation of therapy. Five out of 11 patients exhibited DRMs by UDPS. The issue is whether a therapeutic switch is relevant in these patients by taking into account the identity of the archived resistance mutations. When the archived CTL epitopes were determined on the basis of the HLA alleles, different patterns were observed. Some epitopes were identical to those reported for the reference with the same TA, while others were mutated with a decrease in TA. In 2 cases, an epitope was observed as a combination of subpopulations at entry and was retrieved as a single population with lower TA at success. With regard to immunological stimulation and given the variability of the archived CTL epitopes, we propose a new concept of curative vaccine based on identification of HIV-1 CTL epitopes after prior sequencing of proviral DNA and matching with HLA class I alleles.

## Introduction

HIV-1 infection is a chronic infection with non-stop viral replication leading to a decrease in the number of TCD4 lymphocytes and immunodepression. Viral replication can be limited by antiretroviral drugs of different classes. This reduction in viral replication, which is generally below the threshold of the viral load (VL) commercial assays, is followed by an increase in TCD4 lymphocytes. However, antiretroviral treatment (ART) cannot be stopped even in fully responding patients since various clinical trials have shown that its interruption is followed by the resumption of viral replication. In these patients responding successfully to ART, the next step is viral eradication, otherwise termed viral cure. Various strategies based on pathophysiological data have been proposed and are currently under investigation [1]. For example, it is known that gut lymphoid tissues and the central nervous system are potential reservoirs of the virus and that resting memory T4 cells at the cellular level are latently infected by the virus and are not susceptible to antiretroviral drugs, therefore constituting a reservoir [2]. Viral cure trials to date have ranged from immunological or chemical stimulation of resting T cells to antiviral vaccination, particularly involving TCD8 epitopes, since the importance of the TCD8 cytotoxic response in the decrease in viral replication during the primary infection phase of the disease is well known [3]–[5]. However, it is now clear that these cellular responses and the corresponding attempts at vaccination are dependent on the immunogenetic background of individuals, and mainly on their HLA I alleles [6]–[10].

We investigated HIV-1 infected patients responding successfully to a first-line ART since they are the main target population for attempts at viral cure. These patients are not extensively investigated on a routine basis since they have an undetectable VL. We focused on proviral DNA and addressed two questions. First, are there any resistance mutations to the drugs in proviral DNA, despite the widely held belief that ART is fully successful? Second, by taking into account their HLA I alleles, can the archived viral CTL epitopes be presented to the immunological system of these patients, assuming that replication and release from the archived virus constitute a major part of the emerging viral replication at failure or interruption of ART?

## Results

### Patients and Antiretroviral Treatment (Table 1)

Eleven patients were recruited. The median TCD4 count at initiation of treatment was in agreement with former HIV-1 infections. All were receiving a successful first-line ART 8 months to 9 years after initiation of treatment. No case exhibited any blip during the survey period. All treatments included at least one NRTI/NNRTI drug.

**Table 1:** Antiretroviral treatment and duration, HIV-1 proviral load, viral subtype, resistance mutations in RT, immunogenetics of patients.

Patients	Treatment	Duration of treatment	Proviral load	Subtype	RT mutations (UDPS)	HLA
***A***	FTC TDF LPV/r	4 years	817	B	D67N 0.45%; K70R 5.90%;L101I 1.70%	A*02:06, *03:01;B*44:02, *51:01
***B***		J0	NA		None	A*02:01, *26:01;B*39:01, *40:01
	FTC TDF DRV/r RAL	3 years	3854	B	D67N 0.25%; M184I 23%	
***C***	3TC TDF LPV/r	7 years	177	B	NA	A*01:01, *02:01;B*08:01, *57:01
***D***		J0	NA		none	A*02:01, 24:02;B*27:05, *51:01
	FTC TDF ATV/r	3 years	480	B	D67N 0.74%; K219Q 0.20%;G190E 2.30%	
***E***	FTC TDF RPV	8 months	2334	CRF11_cpx	NA	A*23:01, *68:02;B*07:02, *81:01
***F***		J0	NA		None	A*01:01, *02:01;B*07:02, *51:01
	FTC TDF FPV/r	6 years	789	B	D67N 0.58%; D67E 0.42%;M184I 99.60%; E138G 0.87%;K219R 20%; G190E 12%	
***G***	3TC d4T LPV/r	9 years	572	B	D67N 0.58%; L101I 0.70%;K103R 0.60%	A*03:01, *23:01;B*07:02, *35:01
***H***	3TC TDF LPV/r	9 years	458	B	E138G 0.40%; K219Q 0,03%	A*02:01;B*40:01, *44:02
***I***	AZT 3TC LPV/r (NVP)	9 years	1490	CRF02_AG	M184V 4%; E138G 23%;M230L 20%	A*26:01, *30:02;B*13:03, *18:01
***J***	FTC TDF LPV/r	5 years	139	C	None (NA from aa 219)	A*23:01, *24:02;B*41:02, *53:01
***K***	FTC TDF EFV	6 years	412	B	None	A*24:02, *33:01;B*14:02, *55:01

Proviral load expressed as copies/10^6^ PBMC; NA: not available.

### Molecular Characterization of HIV-1 and Viral Loads (Table 1)

Among 11 HIV-1 isolates, 8 were subtype B and 3 were non-B (CRF02_AG, CRF11_cpx and C). All RNA viral loads (VL) were below the threshold of 50 copies/mL. The proviral DNA load ranged from 139 to 3854 DNA copies/million of PBMC. In all samples, the amount of 2-LTR episomic DNA was below the threshold of 10 copies/million PBMC.

The Gag sequence of one strain (***K***) exhibited 5 stop codons. In all cases, tryptophan amino acid was replaced by a stop codon as a result of a switch from TGG (wild) to TAG or TAA.

### Drug Resistance Mutations (DRMs) Analyzed by Ultradeep Pyrosequencing (UDPS) (Table 1)

With regard to the frequencies of mutant variants above 1%, 3 proviruses exhibited M184I/V mutations between 4 and 99.6%, 2 bore the G190E variants (2.30 and 12% respectively), one had 5.90% K70R and one showed 20% M230L. Two isolates bore two mutations simultaneously: ***F*** with M184I and G190E and ***I*** with M184V plus M230L.

No DRM was observed in the initiation sample from those patients whose viral RNA could be investigated before initiation of ART and who exhibited DRMs in the proviral DNA (***B***, ***D*** and ***F***).

### Nucleotide Variability in Pol Evaluated by UDPS (Figure 1)

In 3 patients, one Pol (RT2 amplicon) region could be studied to evaluate potential nucleotide variability between baseline and the point of success. Two patterns were found: patients ***B*** and ***F*** exhibited different clusters at baseline and at success with a very low variability in each cluster. There was a common sequence at the origin of both clusters. In patient ***D***, there were different clusters at baseline and the point of success was composed of different clusters originating from the initial sequences. Within each cluster, the variability was very low.

### Potential CTL Reactivity against Archived Viral Epitopes According to HLA I Alleles

#### Cross-reactivity to Lipo5 lipopeptides

(Table 2) The ANRS HIV-Lipo5 lipopeptides are composed of the Gag 17–35, Gag 253–284, Nef 66–97, Nef 116–145, and Pol 325–355 peptides. On the basis of the different HLA I alleles and the different available sequences of Gag, Nef and Pol obtained by Sanger sequencing, it was not possible to obtain exhaustive data although some examples can be given.

**Table 2:** Potential CTL reactivity against archived viral epitopes according to HLA I alleles: cross-reactivity to Lipo 5 peptides.

Patients	Viral subtype	HLA alleles	Positions of the lipopeptides	Target analyzed	Epitopes	MHC IC 50
***A***	B	HLA-A*03:01	Pol 325–355	HXB2	AIFQSSMTK	12.04
				Archived	AIFQ*A*SMTK	20.24
***C***	B	HLA-B*08:01	Gag1 253–284	HXB2	EIYKRWII	257.38
				Archived	EIYKRWII	257.38
			Gag2 17–35	HXB2	GGKKKYKLK	31060.99
				Archived	GGKKKYKLK	31060.99
***E***	CRF11_cpx	HLA-B*07:02	Nef1 66–97	HXB2	FPVTPQVPL	13.57
				Archived	FPVKPQVP*V*	30.21
			Nef2 66–97	HXB2	FPVTPQVPLR	20,589
				Archived	FPV*K*PQVP*V*R	10,191
			Nef3 66–97	HXB2	TPQVPLRPM	29.56
				Archived	*K*PQVP*V*RPM	11.17
			Nef4 66–97	HXB2	RPMTYKAAV	5.53
				Archived	RPMTYKAA*F*	4.26
			Nef5 66–97	HXB2	RPMTYKAAL	2.80
				Archived	RPMTYKAA*F*	4.26
***G***	B	HLA-A*03:01	Gag 17–35	HXB2	RLRPGGKKK	158.17
				Archived	RLRRPGGKK*Q*	14,882

Lipo 5 peptides are located in the viral genome and were designed from the HXB2 reference; according to the HLA alleles, we investigated the predicted reactivity against the corresponding epitopes or variants of these epitopes in archived proviral DNA. The MHC IC 50 values provide an estimation of the cross-reactivity between the epitope and the variant. For example, patient G with allele HLA-A*03:01 has archived a variant epitope that is poorly recognized. It is highly doubtful that the corresponding lipopeptide Gag 17–35 HXB2 vaccine lipopeptide will be useful for cross-stimulation.

*A.* The virus was identified as subtype B HIV-1. With an HLA A*03:01 allele, the patient should recognize the Pol 325–355 epitope (AIFQSSMTK), which is one of the Lipo5 peptides designated from the HXB2 HIV-1 subtype B reference. The archived epitope in the provirus exhibited a substitution (AIFQ*A*SMTK) but the presentation with the allele was still excellent (MHC IC50 20.24 versus 12.04).

*C.* The virus was subtype B. With HLA B*08:01, 2 Gag epitopes can be presented according to the HXB2 reference; EIYKRWII with an MHC of 257.38 and GGKKKYKLK with an MHC of 31,060.99 (low affinity). The archived epitopes were identical.

*E* This patient was infected with a CRF11_cpx. With HLA allele B*07:02, 5 epitopes of Nef 66–97 should be recognized. All corresponding archived epitopes were different from those referenced from HXB2 and 4 were efficiently presented while one was not: FPVTPQVPL (MHC 13.57)/FPV*K*PQVP*V* (MHC 30.21); FPVTPQVPLR (MHC 20,589)/FPV*K*PQVP*V*R (MHC 10,191); TPQVPLRPM (MHC 29.56)/*K*PQVP*V*RPM(MHC 11.17); RPMTYKAAV (MHC 5.53)/RPMTYKAA*F*(MHC 4.26); RPMTYKAAL (MHC 2.80)/RPMTYKAA*F*(MHC 4.26).

*G.* The virus was subtype B. According to HLA alleles A*03:01, B*07:02 and B* 35:01, 13 epitopes corresponding to Gag 17–35, Gag 253–284, Nef 66–97 and Pol 325–355 could be evaluated. Most of them exhibited substitutions in the proviral DNA without variation in the MHC. One of them was no longer recognized: Gag 17–35; RLRPGGKKK (MHC 158.17)/RLRRPGGKK*Q* (MHC 14,882).

#### Theoretical presentation of CTL epitopes in the sequenced parts of the archived proviral DNA

Different patterns were noted (Table 3).

**Table 3:** Potential CTL reactivity against viral (RNA) and/or archived proviral DNA epitopes according to HLA I alleles.

Patients	Viral subtype	HLA alleles	Positions of the epitopes	Target analyzed	Epitopes	MHC IC 50
***A***	B	HLA-B*51:01	RT1 42–50	HXB2	EKEGKISKI	36,931
				Archived	EKEGKISKI	36,931
			RT2 128–135	HXB2	TAFTIPSI	2,032
				Archived	TAFTIPS*L*	11,857
***B***	B	HLA-A *02:01	Gag p17 77–85	HXB2	SLYNTVATL	476
				RNA	SL[*F*Y]NT[*I*V]*S*TL	178 to 759
				Archived	SL[Y*F*]NT[V*I*][*S*A][*IVA*T]L	57 to 942
		HLA-B*40:01	Gag p17 92–101	HXB2	IEIKDTKEAL	53
				RNA	[I*M*]*D*[I*V*]KDTKEAL	9,346 to 16,946.
				Archived	I*DV*KDTKEAL	16,946
***G***	B	HLA- A*03:01	RT1 73–82	HXB2	KLVDFRELNK	45
				Archived	KLVDFRELNK	45
		HLA-A*03:01	RT2 158–166	HXB2	AIFKSSMTK	12
				Archived	AIF*Q*CSMTK	16
***H***	B	HLA-B*40:01	Gag p17 92–101	HXB2	IEIKDTKEAL	53
				Archived	I*DV*KDTKEAL	16,946
				Archived	*IDV*KDTKEA*V*	26,985
***F***	B	HLA-A*02:01	RT 179–187	HXB2	VIYQYMDDL	1,946.61
				RNA	VIYQYMDDL	1,946.61
				Archived	VIYQY*I* DDL	855.38

After characterization of HLA alleles, we investigated the CTL epitopes that should be recognized by the patients and have determined the affinity through the MHC IC 50 values. The reference epitopes are from the HXB2 reference. Whether identical or variant, the epitopes were noted in the circulating virus at baseline (RNA) and/or the archived proviral DNA at therapeutic success.

*A.* According to HLA allele A*03:01, the epitopes RT 33–43, RT 73–82, RT 93–101 of HXB2 can be presented with an MHC ranging from 45.58 to 367.82. The epitopes archived in the proviral DNA were identical. With HLA allele B*51:01, 2 epitopes of HXB2, RT 42–50 (EKEGKISKI) and RT 128–135 (TAFTIPSI) could be presented with an MHC of 36,931 and 2,032 respectively. The first archived epitope was unchanged while the second exhibited a substitution (TAFTIPS*L*) with an MHC switch from 2,032 to 11,857.

*B.* We were able to investigate this patient’s viral RNA before the initiation of HAART and his proviral DNA at success. With HLA alleles A*02:01, A*26:01, B*39:01 and B*40:01, 10 epitopes could be analyzed in Gag, Nef and Pol (RT). When we compared the HXB2 reference and viral RNA, 6 epitopes were strictly identical, 2 were mutated (with an increase in MHC) and 2 exhibited double populations at some codons and their corresponding residues.

With HLA allele A*02:01, Gag p17 77–85 (SLYNTVATL) was present with an MHC of 476. The viral RNA presented the epitope SL[*F*Y]NT[*I*V]*S*TL with 4 different combinations and an MHC ranging from 178 to 759. Furthermore, with HLA allele B*40:01, the epitope Gag p17 92–101 IEIKDTKEAL was recognized with an MHC of 53. The corresponding epitopes of the viral RNA exhibited 4 combinations [*I*M]*D*[I*V*]KDTKEAL with an MHC ranging from 9,346 to 16,946.

In the proviral DNA at success, the epitopes were identical to those recorded in the viral RNA but with 2 exceptions: one was mutated (Nef 92–100 presented by the B*40:01 allele) with an MHC increasing from 63 to 198; the other was related to Gag p17 92–101 which exhibits 4 different combinations in the viral RNA. The archived epitope was the one showing the highest MHC (I*DV*KDTKEAL; MHC 16,946).

*G.* For this subtype B and according to the HXB2 reference, 2 epitopes were presented by the HLA allele A*03:01. In the proviral DNA, the first epitope was unchanged while the second was mutated without significant variation in the MHC.

*H.* This patient who is infected with a subtype B HIV-1 is characterized by the HLA alleles A*02:01, B*40:01 and B*44:02. The B*40:01 antigen is able to present the p17 epitope of HXB2 (IEIKDTKEAL) with an MHC of 53. The archived combinations of this epitope (I*DV*KDTKEAL and I*DV*KDTLEA*V*) exhibit MHC values of 16,946 and 26,985 respectively.

**Figure 1:**
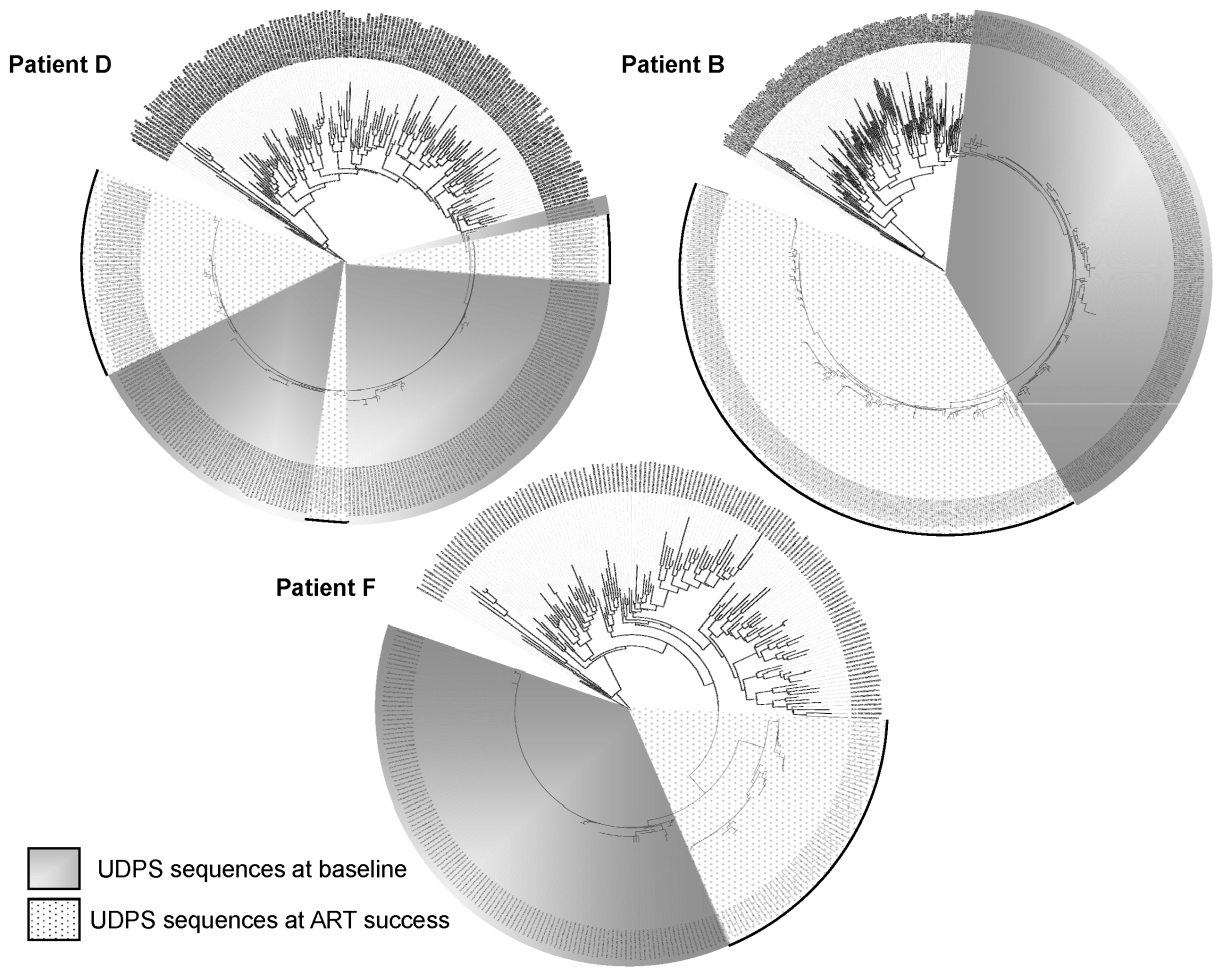
Phylogenetic trees of UDPS sequences (Pol RT2) at baseline and at ART success. Patients D, B and F according to Table 1.

*F.* One epitope matching with HLA allele A*02:01 could be studied by Sanger and UDPS at baseline (RNA) and at success (archived DNA). The HXB2 epitope was VIYQYMDDL and the observed epitope in the viral RNA at baseline was identical with an MHC of 1946.61. At success, the archived epitope was mutated (VIYQY*I*DDL) with an MHC of 855.38. UDPS analysis of the epitope demonstrated high stability of both epitopes at baseline and at success within the subspecies (Figure 1).

## Discussion

In most of these patients fully responding to first-line ART and with a viral load below the VL threshold and no blip, the proviral DNA load was less than 1000 copies/million PBMC. Three patients exhibited a proviral load above 1000 copies for which we have no explanation other than that their RNA and DNA viral loads may have been high at the end of the primary infection step. There was no detectable episomal form of viral DNA and one isolate showed stop codons in the Gag sequence. This fact reflected the G-to-A hypermutation linked to the cellular protein APOBEC [11]. Since the TCD4 values of our patients at initiation of ART indicate that they were not close to primary HIV infection and that they are representative of the main target for HIV cure, i.e. patients at full ART success far from primary infection, it also means that the virus may have evolved between the primary infection and the initiation of ART, and to a lesser extent under ART.

Whereas viral replication was considered to be controlled by ART since initiation and despite the absence of recordable blips, proviral DRMs to NRTIs or NNRTIs were observed in 5 out of the 11 samples. In three of these patients with resistance mutations at success, an RNA sample at baseline did not reveal any resistance mutations. However, our technique is very sensitive and exhibits mutant variants at percentages below 1% [12], while the Sanger technique allows identification of mutant variants, provided that their percentages are above 20% of the subspecies.

We hypothesize that these mutations were selected and archived during the phase of VL reduction after initiation of treatment or during a phase of low-level viremia [13]. These mutations are generally in accordance with the ART (mainly M184V following a regimen containing 3TC or FTC), but this was not the case in two patients (D and F) who never received an NNRTI but exhibited the G190E mutation and who might have been superinfected with viruses bearing resistance mutations.

In the second phase of the study, we analyzed the CTL epitopes of the provirus according to the HLA I alleles that determine the presentation of these epitopes to the cytotoxic CD8 lymphocytes. Considering the lipopeptides which were designed by ANRS (Lipo5) and given the fact that they might have to be identical or very close to the corresponding archived epitopes and able to trigger a significant vaccinal therapeutic response, it is clear that the patterns are highly variable despite these partial results. Some archived epitopes are identical while others are different with a predicted less efficient vaccinal effect. This French therapeutic vaccine was well tolerated in a phase 2 study [14] and shows promise [15], but it is highly doubtful that these generic epitopes designed on the basis of an HXB2 reference will be identical to the archived epitopes.

Regarding the epitopes of the archived provirus and the HLA I alleles, the patterns are also different with epitopes identical to the HXB2 reference and the expected MHC, but also with mutated epitopes that are no longer recognized. Very interesting cases are those where epitopes are recorded as combinations in the viral RNA before initiation of ART and the epitopes finally selected in the proviral DNA are those that are the least efficiently presented. Whether viral evolution can occur together with efficient ART is still unclear. Some studies concluded that it is not possible but they did not focus on individual epitopes, unlike us [16]. Moreover, although preliminary, our results on UDPS sequences of a part of RT show that evolution of the virus does occur at the level of the nucleotide sequence between the plasma and the proviral intracellular compartments.

Our study raises two main issues. First, if the DRMs to the drugs observed in the proviral DNA at success are associated with a low level HIV replication with circulation of resistant variants refueling the reservoirs, it would be relevant to decide on a therapeutic switch in these patients by taking into account the identity of the archived resistance mutations. Among the antiretroviral drugs, integrase inhibitors would be suitable to decrease the archived virus and, if not used as first line, could be used at switch or at treatment intensification [17]. We cannot rule out that these mutations were selected during the reduction of viral replication between the initiation of ART and the first point of VL below the threshold, so it would be interesting to have UDPS data from very recently treated patients to address this issue. The second issue is that, although they were obtained by simulation and not by biological assays (for example, ELIspot) which could hardly be used on a large scale, our results show that curative vaccination with generic epitopes, mainly CTL epitopes, cannot be fully efficient. The epitopes are different from the B reference or are modified when they are archived, as already described [18], and one cannot expect a cross-reaction. Furthermore, these generic epitopes are not systematically suitable for presentation owing to the diversity of class I antigens and corresponding HLA alleles. This is a problem not only for Lipo5 peptides but also for all other generic vaccines based on recombinant viruses or viral DNA [19]. On the other hand, when one first identifies the HLA I alleles and designs potential peptide epitopes on the HXB2 reference, one should be aware that some of these epitopes may be different in the archived provirus. Even the viral RNA reference before initiation of ART can be a decoy because the archived epitopes may be different. If one assumes that the archived proviral DNA is the major origin of viral replication at failure or treatment interruption, we propose that vaccinal epitopes should be selected from the sequenced proviral DNA, in agreement with the HLA alleles of the patients. We plan to extend this study on three different levels: a) on the individual level with a specific analysis of the archived CTL HIV-1 epitopes in one of the main tissue reservoirs, i.e. the gut, and in the long-term cellular reservoir represented by memory resting T cells; b) on the individual level in patients close to primary infection and whose virus is considered to exhibit a lower genomic and antigenic evolution, particularly at positions of CTL epitopes; c) on the population level with recruitment of patients having a different genetic background and infected mainly with non-B HIV-1.

In conclusion, our study opens up therapeutic and vaccinal perspectives in those patients who are considered to be fully responding with ART. A new concept of curative vaccine is proposed where viral CTL epitopes are designated after sequencing of archived proviral DNA and matching with HLA alleles before undertaking vaccination.

## Methods

### Study Patients

Eleven HIV-1 infected patients were enrolled in this study which received authorisation from the « Comité de protection des personnes du Sud Ouest » (DC 2012/48). Written informed consent was obtained from each participant. All were adults responding successfully to a first ART including at least one NRTI and/or NNRTI. Written informed consent was obtained from each participant. The first-line ART period ranged from 8 months to 9 years with undetectable viral load (fewer than 50 copies/ml Roche Ampliprep Cobas Taqman and fewer than 40 copies/ml Abbott) and without intermittent viremia or blip.

At initiation of ART, the median number of TCD4 lymphocytes was 238/uL (range 5–434).

### DNA and RNA Extraction, Quantitation of Proviral DNA and 2-LTR DNA

#### DNA extraction

PBMCs were isolated from EDTA blood samples using Ficoll-Hypaque gradient centrifugation. After separation, PBMCs were pelleted by centrifugation into 2.106 to 10.106 aliquots and cell pellets were kept frozen at −80°C until the analysis. Total DNA (including integrated HIV-1 DNA and episomal HIV-1 DNA) was extracted from patients’ PBMCs using the MagNAPure Compact kit (Roche Diagnostics, Mannheim, Germany) according to the manufacturer’s protocol.

#### RNA extraction

Frozen (−80°C) EDTA plasma from patients at initiation of ARV therapy was used for viral RNA extraction, which was performed using the MagNA Pure LC Total Nucleic Acid Isolation-High Performance kit with the MagNA Pure LC system (Roche Diagnostics).

#### Total HIV-1 DNA quantification

Total HIV-1 DNA was amplified by quantitative real-time PCR using the light Cycler Instrument (Roche Diagnostics). Amplification was performed in a 20 µl reaction containing 1 X Light Cycler Fast Start DNA Master Hybridization probes (Roche Diagnostics), 3 mM MgCl2, 500 nM forward primer LTR152 (5′-GCCTCAATAAAGCTTGCCTTGA-3′ and 500 nM reverse primer LTR131 (5′-GGCGCCACTGCTAGAGATTTT-3′) located in a long terminal repeat (LTR) region with low variability and 50 nM fluorogenic hybridization probe LTR1 (5′6FAM-AAGTAGTGTGTGCCCGTCTGTT(AG)T(GT)TGACT-3′Tamra). After an initial denaturation step (95°C for 10 min) total HIV-1 DNA was amplified for 45 cycles (95°C 10 sec, 60°C 30 sec) followed by 1 cycle at 40°C for 60 sec. The copy number of total HIV-1 DNA was determined using the 8E5 cell line. The 8E5/LAV cell line used as a standard curve was derived from a CEM cellular clone containing one single, integrated and defective (in pol open reading frame) viral copy genome that is constitutively expressed. Five to 5.104 copies of 8E5 DNA were amplified. Results were expressed as copy number of total HIV-1 DNA per 106 PMBC.

#### 2-LTR DNA circle quantitation

The 2-LTR DNA circles were amplified with primers, HIV-F and HIV-R1, spanning the LTR-LTR junction. Two-LTR junctions were amplified with 30 ng of total cell DNA. The reaction mixture contained 12.5 µl of SYBR Green qPCR master mix 2X (Fermentas, St Remy les Chevreuses, France), 300 nM forward primer HIV-F (5′- GTGCCCGTCTGTTGTGTGACT-3′), 300 nM reverse primer HIV-R1 (5′- ACTGGTACTAGCTTGTAGCACCATCCA-3′) in a final volume of 25 µl; the amount of 2-LTR circles DNA was determined in a MyiQ real time PCR thermocycler (Biorad, Marnes-La-Coquette, France). The PCR conditions were: 95°C for 3 min, (95°C for 10 sec; 60°C for 30 sec; 72°C for 30 sec) for 42 cycles, 60°C for 10 sec by increasing set point temperature after cycle 2 by 0.5°C for 80 cycles. The copy numbers of 2-LTR DNA circles were determined by reference to a standard curve prepared by amplification of quantities ranging from 10 to 106 copies of cloned DNA. The quantification results were expressed as copy numbers per 106 PBMC from patients. The lowest limit of detection of 2-LTR DNA by using the SYBR Green qPCR was 10 copies of 2-LTR/assay.

### PCR Amplification of Gag, Nef and Pol Regions

Epitopic regions of Gag and Nef were amplified from DNA and/or RNA extracted previously using primers described in Table 4 and AmpliTaq Gold with GeneAmp Kit (Applied Biosystem, Foster City, CA). The epitopic region of RT was amplified using primers described in Table 4 and FastStart Taq DNApol HiFi (Roche).

**Table 4:** Primers used for Gag, Nef and Pol amplification.

		sequence 5′-3′	HXB2 genome position
GAG	First PCR 1^st^ amplicon		
	Primer 5′	GACTAGCGGAGGCTAGAA	764–781
	Primer 3′	TTTGGTCCTTGTCTTATGTCCAGA	1635–1658
	First PCR 2^nd^ amplicon		
	Primer 5′	ATAATCCACCTATCCCAGTAGGAGAAATT	1544–1572
	Primer 3′	ATGCTTTTATTTTTTCTTCTGTCAATGGC	2621–2650
	Second PCR 1^st^ amplicon		
	Primer 5′	GACTAGCGGAGGCTAGAA	764–781
	Primer 3′	GTTCTAGGTGATATGGCCTGATG	1219–1241
	Second PCR 2^nd^ amplicon		
	Primer 5′	CACCTAGAACTTTAAATGCATGGGT	1232–1256
	Primer 3′	TTTGGTCCTTGTCTTATGTCCAGA	1635–1658
	Second PCR 3^rd^ amplicon		
	Primer 5′	ATAATCCACCTATCCCAGTAGGAGAAATT	1544–1572
	Primer 3′	AGGGGTCGTTGCCAAAGA	2264–2281
	Second PCR 4^th^ amplicon		
	Primer 5′	TCAGAGCAGACCAGAGCCAACAGCCCCA	2136–2163
	Primer 3′	AATGCTTTTATTTTTTCTTCTGTCAATGGC	2621–2650
NEF	First PCR		
	Primer 5′	GCCACAGCCATAGCAGTAGCTGAGGGG	8673–8699
	Primer 3′	CCAGTACAGGCAAAAAGCAGCTGCTTATA	9511–9540
	Second PCR		
	Primer 5′	CCTAGAAGAATAAGACAGGGCTTGGAAAG	8754–8782
	Primer 3′	ACGCCTCCCTGGAAAGTCCCCAGCGG	9443–9468
POL	First PCR		
	Primer 5′	AGTAGGACCTACACCTGTCA	2480–2499
	Primer 3′	CTGTTAGTGCTTTGGTTCCTCT	3399–3420
	Second PCR 1^st^ amplicon		
	Primer 5′	TTGGTTGCACTTTAAATTTTCCCATTAGCCCTATT	2530–2564
	Primer 3′	CTTTCCATCCCTGTGGAAGCACATT	2988–3012
	Second PCR 2^nd^ amplicon		
	Primer 5′	GAAAATCCATACAATACTCCAGTATTTGC	2706–2734
	Primer 3′	CTATGCTGCCCTATTTCTAAGTCAGAT	3119–3145
	Second PCR 3^rd^ amplicon		
	Primer 5′	CTRGATGTGGGTGATGCA	2874–2891
	Primer 3′	CNYTATAGGCTGTACTGTCC	3284–3265

### Gag, Nef and Pol Sanger Sequencing

PCR products were sequenced on both strands using an Applied Biosystems 3500xls Dx Genetic Analyzer and primers from the second PCR. The sequences of the study are available in GenBank under accession numbers KC899326–KC899346.

### RT Ultra-deep Pyrosequencing

RT UDPS was performed using the Roche GS Junior equipment. Amplicons previously obtained, purified and quantitated were pooled at equimolar concentrations. Clonal amplification on beads (EmPCR) was performed using the 454 Life Science reagents that enable bidirectional sequencing, composed of 30 cycles of PCR amplification. DNA-containing beads were recovered and UDPS was performed on the GS Junior sequencer (454 Life Sciences; Roche). UDPS generated a median of 11.000 sequence reads per sample. These reads were analyzed using the Amplicon Variant Analyzer software, 454, Roche. The UDPS results of the study are available in GenBank under accession number SRA073324.

### HLA Class I Typing

Genomic DNA was extracted from the frozen white blood cell pellets and quantitated as described above. Intermediate-to-high resolution was performed by reverse Polymerase Chain Reaction-Sequence Specific Oligonucleotide (PCR-SSO) hybridization using the LuminexH flow beads LabTypeH assay (InGen, Chilly-Mazarin, France) for the A and B loci. Allelic ambiguities were solved with PCR-Sequence Specific Primer (SSP) amplification using Olerup assays (BioNoBis, Montfort L’Amaury, France). The manufacturers’ recommendations were strictly followed. Allele assignment was performed by comparison with the official nomenclature and validated by the WHO committee for HLA system factors.

### Immune Recognition Tools

The viral epitopes considered for the study were those from the Los Alamos database. Recognition between the HLA groove and the peptides or their variants was predicted using the immune epitope database (www.immuneepitope.org). We evaluated the affinity of the epitopes for the MHC molecules with the MHC IC50 (nM) value. Small values are associated with better binders. A value of 500 nM is often used as the threshold between binders and non-binders.
